# An exploratory study of the variables impacting preterm birth rates in New Mexico

**DOI:** 10.1186/1471-2393-12-53

**Published:** 2012-06-21

**Authors:** Kara M Gwin, Ronald Schrader, Kimberley Peters, Armida Moreno, Kristina W Thiel, Kimberly K Leslie

**Affiliations:** 1 Department of Obstetrics and Gynecology, University of New Mexico, Albuquerque, NM, USA; 2 Department of Internal Medicine, University of New Mexico, Albuquerque, NM, USA; 3 Department of Health of the State of New Mexico, Albuquerque, NM, USA; 4 Department of Obstetrics and Gynecology, University of Iowa, Iowa City, IA, USA; 5 Holden Comprehensive Cancer Center, University of Iowa, Iowa City, IA, USA

**Keywords:** Preterm birth, Prenatal care, Education, Ethnicity, Maternal age

## Abstract

**Background:**

Preterm birth (PTB) is a substantial health problem that accounts for significant infant morbidity and mortality and poses an economic burden to both individuals and the state of residence. The goal of this study was to identify maternal risk factors for PTB in New Mexico, a poor state with a unique ethnic background, in order to identify populations at increased risk that would benefit from intervention.

**Methods:**

This was a cross-sectional retrospective exploratory analysis of 377,770 singleton live births in the state of New Mexico from 1991-2005. Gestational age of less than 37 weeks was defined as PTB. The Kotelchuck Index was used as a measure for level of prenatal care described as inadequate, intermediate, adequate, and intensive.

**Results:**

Of the live births analyzed, 28,036 of these were preterm (7.4%). Overall the PTB rate rose at a rate of 0.18% per year from 1991-2005. Among patients with medical risk factors, the absence of prenatal care was associated with higher odds for PTB as compared to adequate prenatal care. Other risk factors were unmarried status, education less than high school, tobacco/alcohol use, black, Asian, and white Hispanic ethnicity, and the presence of one or more medical risk factors. Statistically significant protective factors for PTB were age 25-29, education surpassing high school, and Native American race.

**Conclusions:**

This study identified several factors that correlate with increased PTB in New Mexico, in particular ethnicity and level of prenatal care. The finding that Native American patients have a lower PTB rate compared to other groups, even though this group is traditionally one of low socioeconomic status in New Mexico, signifies that other factors yet to be identified affect PTB.

## Background

Preterm birth (PTB), defined as birth before 37 weeks gestational age, is a significant health problem throughout the world. In the US, disorders associated with prematurity account for the leading cause of death during the neonatal period (birth to 28 days) and is second only to congenital malformations as the major cause of death during infancy (birth to one year) [1]. According to the March of Dimes Premature Birth Report Card, one in eight infants were born preterm in 2010 in the US. By comparison, the PTB rate in New Mexico rose from 10.8% in 2004 to 12.3% in 2008. Furthermore, prematurity-associated disorders were the leading cause of death in infancy in New Mexico in 2004 [2]. In order to begin to combat this significant problem at both the national and state levels, a clear elucidation of the causes of PTB is needed.

The causes of preterm labor are multifactorial and continue to be a focus of research. In about 20% of cases, preterm labor is induced due to complications of pregnancy (iatrogenic) [3]. Some examples of such complications are preeclampsia, the related HELLP syndrome, placental abruption and intrauterine growth restriction [4]. However, the majority of preterm labor occurs spontaneously. Although some factors that predispose to preterm labor and/or birth have yet to be discovered, some risks have been identified. Women with a previous spontaneous preterm delivery have a 2.5-fold greater incidence of PTB as compared to multiparous women with no prior PTB [5]. In observational studies, lack of prenatal care has also been associated with higher rates of PTB [6,7], though similar results were not obtained in randomized trials in which a high level of prenatal care was administered to high-risk patients [8]. Low levels of education, extreme maternal age, and single marital status have been classified as risk factors for PTB [9]. Finally, maternal race has also been shown to be a factor in rates of preterm delivery, with a majority of research in the US focusing on the approximately two- to three-fold greater risk of PTB among black women as opposed to white women [9,10].

With regards to race and ethnicity in particular, black women have the highest percentage of preterm births, with an average rate of 17.8% in 2003 as compared to 11.3% for non-Hispanic whites [10]. The most remarkable increases in PTB in the US from 2001 to 2003 were among non-Hispanic whites (2.73%), Hispanic (2.59%), and Native American (3.05%) ethnicities as compared to the national increase of 1.65% [10,11]. This information is of particular interest for this study given that non-Hispanic whites account for 43.5% of New Mexico residents, 41.5% are Hispanic, 11% are Native American, and only about 4% of the population are black or of Asian descent [12]. Few studies have evaluated the relationship between Native American ethnicity and PTB, with one study showing that this group has increased maternal risk factors (e.g., tobacco and alcohol use, hypertension), decreased utilization of prenatal care and a 32.5% higher PTB rate as compared to non-Hispanic whites [13]. While variables other than race/ethnicity may contribute to the increased PTB rate in Hispanics and Native Americans, such as socioeconomic disparities, it is important to understand if the national trends of PTB among different ethnicities are replicated in New Mexico.

In addition to ethnic and racial distribution, New Mexico has a high proportion of other potential socioeconomic risk factors for PTB. First, the median household income in 2005 ranked 44^th^ of 50 nationally, and in that same year 18.5% of the population lived below the poverty level. The state also ranked only 41^st^ for residents >25 years old with a high school diploma, demonstrating a level of education well below the US mean. Finally, a recent retrospective study of utilization of prenatal care in New Mexico 1989-1999 demonstrated that low-income mothers had significantly fewer prenatal visits than high-income mothers [14]. The aim of this observational study was to identify sub-populations at increased risk for PTB in New Mexico and to understand if any specific maternal factors, particularly socioeconomic variables or level of prenatal care, strongly correlate with incidence of PTB.

## Methods

This was a cross-sectional study. Birth data from 1991-2005 were obtained from the New Mexico Department of Health Bureau of Vital Records and Health Statistics. The data were ascertained from birth certificates from all hospitals and birth centers in the state. The completeness and the accuracy of the data were reviewed and audited by the Department of Health, the State of New Mexico. This study was reviewed by the University of New Mexico Human Research Review Committee and was deemed to be exempt. Unless specified, all the reported results are adjusted.

The time frame of 1991-2005 was chosen because birth certificate data were collected by a consistent method in this database beginning in 1991; information collected before 1991 included different variables and was not equivalent. Extending the observation period over 15 years allowed us to determine how PTB trended over time. From the 423,612 births recorded during this 15 year time frame, we determined the rate of PTB (which was defined as birth at a gestational age of less than 37 weeks zero days but greater than 22 weeks zero days), the level of prenatal care utilization, maternal ethnicity, maternal age, maternal education level, marital status, tobacco or alcohol use during pregnancy, county of maternal origin, birth year, and high vs. low risk pregnancy (Table 1). Gestational age was based on last menstrual period, first examination, and ultrasound, when available, and was consistent with the physician’s best estimate of the due date. This variable is termed estgest, which is distinguished from a second variable in the dataset, calgest, which was based solely on the last menstrual period. Calgest was not consistently filled out and was missing in 15% of the dataset, whereas estgest was missing in only 0.02% of the dataset. Level of prenatal care utilization was calculated utilizing the Kotelchuck index (also adequacy of prenatal care utilization/APNCU) [6] since this index is the most widely reported measure of prenatal care and believed to be the most comprehensive. The Kotelchuck index incorporates both the timing of initiation of prenatal care (segregated into two month intervals instead of by trimester as in the GINDEX, another popular index) and visit index (compared the actual number of visits between initiation of prenatal care and delivery to the American Congress of Obstetrics and Gynecology (ACOG) recommended expected number of visits, whereas the GINDEX only calculates the total number of visits) [15]. Maternal ethnicity was categorized as Asian, White non-Hispanic, White Hispanic, Native American, Black or other. Mixed race was categorized as other. White Hispanic excludes persons of Native American, Asian, or Black descent. Asian includes Asian and Pacific Islander. Facility types were hospital, birth center, home or other. For simplicity of analysis, maternal age was divided into less than 15, 15-19, 20-24, 25-29, 30-34, 35-39 and greater than 40. We used maternal education level as a surrogate of maternal socioeconomic status (SES), since maternal household income was not available from the database utilized and because it has been previously theorized to be a good indicator of SES [16]. Maternal education level was divided into less than 9 years of education, 9-12 years of education, between 13 and 16 years of education, and greater than 16 years of education. In New Mexico this would be equivalent to no high school (<9), some high school (9-12), at least some undergraduate education (13-16), and at least some graduate level education (>16). Amount of tobacco or alcohol consumption was not available through the database, so these data were analyzed as simple dichotomous variables. A pregnancy was considered high risk if there was a maternal condition that could have contributed to an increased risk for PTB (diabetes, eclampsia, pregnancy induced hypertension (PIH), chronic hypertension (cHTN), oligohydramnios, incompetent cervix, previous premature delivery, Rh sensitization, pulmonary, cardiac, and renal conditions, and other medical risk factor not otherwise specified). Subanalysis of iatrogenic vs. spontaneous PTB was not performed because these data were not reliably recorded on the birth certificates. Given that this analysis was focused on maternal risk factors, fetal conditions that could have contributed to PTB for reasons other than maternal factors, such as congenital and chromosomal anomalies, were excluded. For the same reason, we excluded pregnancies complicated by multiple gestations. With the exclusions, data from 377,770 individual births were analyzed using SAS Version 9.1. Logistic regression was used to perform this multivariable analysis. Linear trend was detected by treating the year as a continuous variable and examining the coefficient of that variable by logistic regression. Based on conflicting data from observational studies and randomized trials [6-8], the interaction between maternal risk and level of prenatal care was also included in the logistic regression model. Adjusted odds ratios, confidence intervals, and p values for risk factors relating to PTB are presented in Table 2. Multiple records of singleton births per unique woman were included because it was not possible to determine from the birth records if the woman had a previous birth in the time period of the study. To account for this potential maternal clustering, we have added an additional analysis as a way to demonstrate the maximum impact that multiple births to a single woman would have had on our study. We estimated an upper bound on the average cluster size based on the frequency of distribution of the variable “birth order of all live births delivered by mother” (1 = this event is first birth, etc.), which estimated that 24% of clusters are of size 1, 38% of size 2, 27% of size 3, and 5% of size 4 or more. We used a Variance Inflation Factor of 2, which estimates an average cluster size of 2 and within cluster correlation ρ = 1. Thus all standard errors of log(OR) from logistic regression were multiplied by √2 to produce the confidence intervals and p-values for adjusted OR.

**Table 1:** Demographics, unadjusted odds ratios, and excluded records, New Mexico 1991-2005

**No. of records total 1990-2005: 451742**				
**Minimum no. in a year (1997): 27484**				
**Maximum no. in a year (2005): 29456**	***Frequency***	***% of total***	***% Preterm after Exclusions***	***Unadjusted Odds Ratio***
**Ethnicity**				
Asian	5889	1.3	8.66	0.97
Other	254	0.06	12.7	1.49
White Hispanic	219893	48.68	9.26	1.049
Native American	63115	13.97	7.77	0.869
Black	8378	1.85	12.7	1.49
White Non-Hispanic	154213	34.14	8.92	Reference
Missing	26673	5.9	20	6
Inadequate	107576	23.81	8.66	2.28
Intermediate	80289	17.77	3.63	0.90
Adequate	139993	30.99	4	Reference
Intensive	97211	21.52	18.01	5.27
**Maternal Age**				
< 15	1446	0.32	14.43	1.90
15-19	75920	16.81	9.76	1.22
20-24	135445	29.98	8.63	1.06
25-29	114764	25.4	8.16	Reference
30-34	79154	17.52	8.91	1.10
35-39	36438	8.07	10.46	1.31
> = 40	8307	1.84	12.4	1.59
Missing	268	0.06		
**Maternal Education Level (# of years)**				
< 9	27216	6.02	8.75	0.96
9-12	259107	57.36	9.11	Reference
13-16	120997	26.78	8.33	0.91
> 16	30040	6.65	8.26	0.90
Missing	14382	3.18		
**Facility**				
hospital	445529	98.62	9.05	Reference
birth center	1192	0.26	1.68	0.17
home	4131	0.91	4.58	0.48
other	890	0.2	12.97	1.50
**Maternal Marital Status**				
Missing	4	0		
Married/inferred married	253396	56.09	8.42	Reference
Unmarried/inferred unmarried	198342	43.91	9.72	1.17
**Maternal Tobacco Use**				
No	399904	88.52	8.5	Reference
Yes	45737	10.12	11.84	1.45
Missing	6101	1.35	20.49	2.77
**Maternal Alcohol Use**				
No	435079	96.31	8.71	Reference
Yes	9540	2.11	13.02	1.57
Missing	7123	1.58	20.84	2.76
No	376322	83.30	7.25	Reference
Yes	75420	16.7	17.69	2.75
Individual high risk factors*				
Lung	7158	1.58		
Cardiac	1996	0.44		
Diabetes	15686	3.47		
Eclampsia	3881	0.86		
Hydramnios/oligohydramnios	7477	1.66		
Chronic hypertension	3634	0.8		
Pregnancy-induced hypertension	21189	4.69		
Incompetent cervix	703	0.16		
Previous preterm infant	4215	0.93		
Rh sensitization	6032	1.34		
Tocolysis	15749	3.49		
**Overall Preterm (estgest < 37)**				
No	410346	90.84		
Yes	40550	8.98		
Missing estgest value	846	0.19		
Total exclusions for anomalies**	9229	2.04		
**Multiple births or missing information on number born**	9950	2.20		
Out of state resident***	10926	2.42		

* More than one risk factor may be present per unique record.

** Excluded anomalies: Anecephalus, Spina Bifida, Hydrocephalus, Microcephalus, Other Central Nervous System Anomaly, Heart Malfunction, Other Circulatory/Respiratory Anomaly, Rectal Atresia/Stenosis, Other Gastrointestinal Anomaly, Undescended Testicle, Hypospadias and Epispadias, Other Malformed Genitalia, Renal Agenesis, Other Urogenital Anomaly, Cleft Lip, Cleft Palate, Polydactyly, Syndactyly, Other Reduction Deformities, Congenital Dislocation of Hip, Other Musculoskeletal Integration, Down's Syndrome, Chromosomal Anomaly, Other Congenital Anomaly, and Undesignated Congenital Anomaly.

***This variable was included because of the large number of foreign nationals in New Mexico.

**Table 2:** Adjusted odds ratios for risk of PTB by demographic and perinatal characteristics, New Mexico, 1991-2005

	***Adjusted Odds Ratio***	***95% Confidence Interval***	***p value***
**Maternal Age**			
*Reference age = 25-29*			
Age Group			
<15	1.9	1.47-2.46	<0.0001
15-19	1.22	1.15-1.30	<0.0001
20-24	1.07	1.02-1.13	0.0059
30-34	1.09	1.03-1.15	0.0037
35-39	1.25	1.17-1.35	<0.0001
>40	1.34	1.18-1.52	<0.0001
**Maternal Education Level**			
*Reference education level = 9-12 years*			
Number of years			
<9	0.995	0.92-1.07	0.91
13-16	0.89	0.85-0.93	<0.0001
>16	0.82	0.76-0.89	<0.0001
**Maternal Ethnicity**			
*Reference ethnicity = White Non-Hispanic*			
Asian	1.23	1.05-1.43	0.0087
Other	1.49	0.76-2.92	0.2452
White Hispanic	1.05	1.00-1.10	0.0372
Native American	0.88	0.82-0.95	0.0016
Black	1.37	1.22-1.54	<0.0001
**Normal Risk Pregnancy and Level of Prenatal Care**			
*Reference level of care = Kotelchuck adequate*			
Kotelchuck Index			
None	4.92	4.53-5.34	<0.0001
Inadequate	2.16	2.02-2.30	<0.0001
Intermediate	0.93	0.85-1.01	0.0728
High	4.95	4.66-5.25	<0.0001
**High Risk Pregnancy and Level of Prenatal Care**			
Kotelchuck Index			
None	14.66	12.92-16.64	<0.0001
Inadequate	4.78	4.34-5.26	<0.0001
Intermediate	2.59	2.26-2.97	<0.0001
Adequate	2.31	2.08-2.56	<0.0001
High	8.42	7.83-9.05	<0.0001

## Results

**Figure 1:**
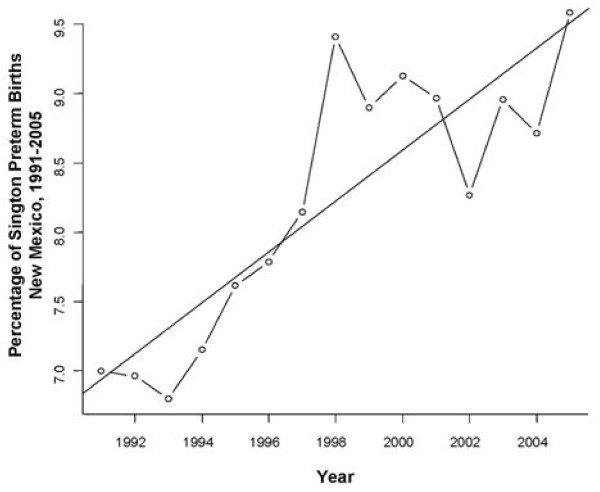
**Rate of PTB among singleton deliveries in New Mexico, 1991-2005.** The rate of PTB in New Mexico between 1991-2005 was assessed by treating year as a continuous variable and examining the coefficient of that variable by logistic regression. A linear trend of increasing PTB over time was observed.

**Figure 2:**
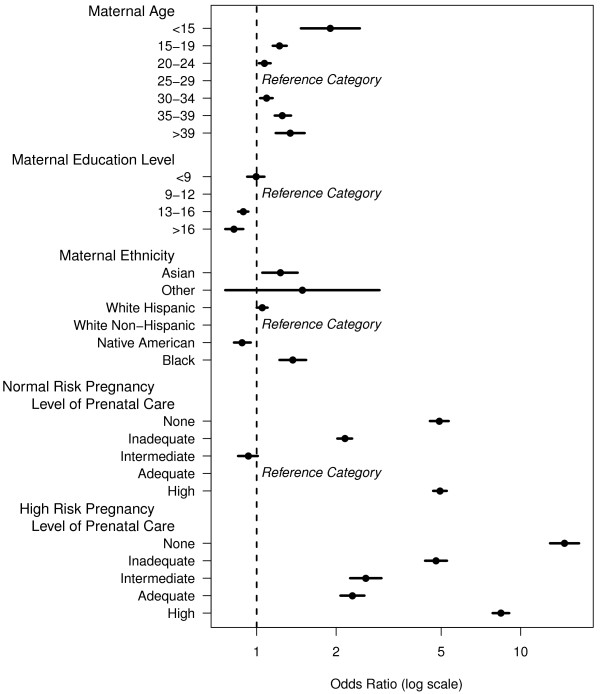
**Adjusted odds ratio of PTB in New Mexico, 1991-2005.** Maternal reference age = 25-29. Maternal reference education level = 9-12 years. Maternal reference ethnicity = White Non-Hispanic. The age group with the lowest odds for PTB in the state of New Mexico is 25-29 years. The odds for PTB were higher among younger and older parturients compared to women 25-29 years of age. Maternal education beyond 12 years was associated with significantly decreased odds of PTB in New Mexico. The ethnic group with the highest odds for PTB was Blacks and the lowest was Native Americans.

In this study, 377,770 total births were included for analysis. Of these, 28,036 births were classified as preterm, which was 7.4% of births evaluated. Overall, PTB had a statistically significant linear trend upward of 0.18% per year over the 15 year period (Figure 1, p <0.00004). Women who had missing, inadequate and intensive levels of prenatal care per the Kotelchuck index had increased odds of having a preterm baby as compared to women with an adequate level of prenatal care (Table 2). Interestingly, patients with an intermediate level of prenatal care had slightly reduced odds for PTB (Figure 2, Table 2).

Not surprisingly, those women with medical risk factors for PTB had statistically significantly higher odds of having a preterm baby for all levels of prenatal care than those without medical risk factors (Table 2). The effect of no prenatal care was particularly severe among women with medical risk factors.

Women of Asian, white Hispanic, and black ethnicities had increased odds for PTB compared to white non-Hispanic mothers (Table 2). Being of Native American ethnicity conferred a statistically significant protective effect on odds for PTB (Table 2). Women of all other ethnicities did not differ significantly compared to white non-Hispanics (Figure 2, Table 2).

Maternal age of 25-29 conferred the lowest odds for PTB. The odds increased significantly on either side of the maternal age extremes. Age of less than 15 years conferred the greatest odds for PTB, with an odds ratio almost double that of mothers giving birth at ages 25-29 (Figure 2, Table 2).

Having greater than 12 years of education was a statistically significant protective factor for PTB. Less than 9 years of education as compared to 9-12 years did not confer any statistically significant benefit or odds for PTB (Figure 2, Table 2). We also examined whether education levels had differing benefits across ethnicities. However, we found that the benefits of education in reducing PTB were consistently seen regardless of the ethnic group evaluated (data not shown).

Unmarried mothers had 14% increased odds for PTB (OR = 1.14; 95% CI = 1.10-1.17; p <0.0001) as compared to women who were married at the time of parturition. Additionally, alcohol and tobacco use during pregnancy both increased the odds of PTB by 37% (95% CI = 1.32-1.42; p <0.0001) for tobacco use and 30% (95% CI = 1.21-1.41; p <0.0001) for alcohol use.

## Discussion

In this analysis of deliveries in New Mexico from 1991-2005, many risk factors for PTB were elucidated. We found that even excluding fetal conditions that could predispose to PTB, almost 8% of babies were born at an early gestational age. Additionally, the rate of PTB rose in New Mexico over 1991-2005, and the rise was statistically significant. This is consistent with trends across the country [3,10,17,18]. Those factors that conferred greater risk included the following: (1) having too little prenatal care, (2) a high risk pregnancy complicated by maternal risk factors, (3) being of Asian, white Hispanic or black ethnicity compared to non-Hispanic white, (4) extremes of maternal age, either young or old, (5) less maternal education, (6) unmarried mothers, and (7) alcohol or tobacco use. Protective factors in our analysis were (1) having intermediate or adequate levels of prenatal care, (2) having no medical risk factors, (3) being of Native American ethnicity, (4) maternal age 25-29, (5) having more than a high school education, (6) being married, and (7) abstaining from alcohol and tobacco use during pregnancy.

Women with a lack of or inadequate prenatal care as defined by the Kotelchuck index had significantly increased odds of having a PTB. Among women with normal risk pregnancies, an intensive level of prenatal care was associated with higher odds for PTB as compared to intermediate care. However, the likely reason for this unexpected trend is that these “normal risk” patients had a complication that was not reported on the birth certificate data that resulted in intensive prenatal care. Having a high risk pregnancy increased the risk of PTB, similar to findings from numerous other studies [9]. However, among women with high risk pregnancies, an intensive level of prenatal care correlated with a decreased the risk for PTB as compared to no prenatal care, which is consistent with several other observational studies [6-8]. Our observation that high risk pregnancies that received intensive prenatal care had increased odds of PTB may simply reflect the severity of complications associated with those pregnancies. It should be noted, however, that similar results were not obtained in randomized trials, suggesting that other factors secondary to prenatal intervention affect PTB rates [8].

Blacks, Asians, and Hispanics had an increased risk of PTB compared with White non-Hispanics. The increased risk for PTB among black women is not surprising given the large number of confirmatory studies previously published [19-22]. Hispanics did have a statistically significant increase in risk for PTB in this study, but it was very slight, with an increased risk of only 5%. Interestingly, recent immigrants have been found to have a lower incidence of preterm birth as compared to immigrants living in the US for greater than five years [23]. Though our study did not segregate based on immigration status or number of years in the US, it would be of interest to understand how this variable affects preterm birth rates among Hispanics in New Mexico since they comprise nearly half of the total population in the state.

More interesting, perhaps, is the protective effect of Native American ethnicity on PTB. This is one of the major contributions of our study since there have been few comparative studies on PTB in the Native American population [13,14,24]. However, a majority of these studies found that Native Americans have decreased PTB rates as compared to other high-risk races/ethnicities such that rates are similar to non-Hispanic whites [14,24]. For example, a recent study by Schillaci found no correlation with level of prenatal care and incidence of PTB. This lack of trend has been observed with other high-risk variables in the Native American population, including poverty-level income, single marital status, and a low level of maternal education [14,24]. Future studies are necessary to identify causal factors that decrease PTB incidence in Native Americans in New Mexico and may be related to behavioral or psychosocial factors that are specific to this ethnicity and not available for analysis in this study. It should be noted that prenatal care for Native Americans in New Mexico is universally available through the Indian Health Service, and it is also possible that such access contributes to the reduced preterm birth rate in this population.

Though information was available on a large number of patients over a 15-year span, a limitation of this study, as with all research that depends upon data collected from birth certificates, was that not all clinical information was recorded. For example, birth certificates do not record whether the PTB was medically indicated for maternal complications. Because information about multiple births per unique woman was not available in the dataset, we did not limit the analysis to one record per unique woman but we did include a variance inflation factor to account for the maximum impact the clustering might have had. Also, we were not able to refine this analysis for the variables of parity or medical or surgical interventions to prevent or treat preterm labor, such as cerclage or tocolysis. Such indicated deliveries certainly contributed to the increased numbers of PTBs among women with risk factors which we found in this study. This study used the date of the last menstrual period, first examination, and ultrasound, when available, as the primary variable to define gestational age (termed estgest variable, which is consistent with the physician’s best estimated gestational age). This value was used because the calculated gestational age based on last menstrual period alone (calgest) was not available for many records and because the estgest variable is more reliable. It should be noted, however, that the increased use of ultrasound to estimate gestational age, which was included in estgest, over the span of the study could have provided a more accurate measure for cases that occurred later in the time period. Next, though maternal complications were available for inclusion in the analyses, previous studies have demonstrated that this information as well as behavioral risk factors (e.g., smoking/alcohol use) may be under- or over-reported on birth records [25]. Similarly, it is possible that medical conditions that are classified as high risk factors for PTB were under-reported, particularly for those women with an absence of or inadequate prenatal care. However, among those women without recorded risk factors, iatrogenic preterm delivery prior to 37 weeks is likely to be quite low, and we do not believe that iatrogenic delivery in low risk patients skewed the data. Thus, we surmise that the majority of PTBs in low risk women were spontaneous, though future prospective analyses will be needed to confirm this assumption.

## Conclusions

In conclusion, we have identified many risk factors for PTB among New Mexico women, including those at the extremes of age, those of lower socioeconomic status, those of Hispanic, Asian, or black ethnicity and those with underlying medical risk factors. Since our data indicate that adequate prenatal care correlates with a lower risk of PTB for normal risk pregnancies, and lack of prenatal care was associated with increased probability of PTB for high risk pregnancies, we propose that adequate levels of prenatal care have the potential to decrease incidence of PTB. However, much remains unknown with respect to PTB. By further study and increased provider vigilance, it is hoped that we can curb this significant health problem and improve pregnancy and neonatal outcomes.

## Competing interests

The authors declare no competing financial or non-financial interests.

## Authors’ contributions

KMB designed the study, collected and analyzed data, and drafted the manuscript. RS analyzed data, performed statistical analyses, and helped draft the manuscript. KP assisted in study design and procurement of data. AM assisted in data procurement and analysis. KWT analyzed data and helped draft the manuscript. KKL conceived of the study, participated in its design and coordination, and helped to draft the manuscript. All authors read and approved the final manuscript.

## Pre-publication history

The pre-publication history for this paper can be accessed here:

http://www.biomedcentral.com/1471-2393/12/53/prepub
